# Performance of machine learning models to forecast PM10 levels

**DOI:** 10.1016/j.mex.2024.102557

**Published:** 2024-01-05

**Authors:** Lakindu Mampitiya, Namal Rathnayake, Yukinobu Hoshino, Upaka Rathnayake

**Affiliations:** a Water Resources Management and Soft Computing Research Laboratory, Millennium City, Athurugiriya 10150, Sri Lanka; b Department of Civil Engineering, Faculty of Engineering, The University of Tokyo, 1 Chome-1-1 Yayoi, Bunkyo City, Tokyo 113-8656, Japan; c School of Systems Engineering, Kochi University of Technology, Tosayamada, Kami, Kochi 782-8502, Japan; d Department of Civil Engineering and Construction, Faculty of Engineering and Design, Atlantic Technological University, Sligo F91 YW50, Ireland

**Keywords:** Air quality, Ensemble model, Forecasting, PM10 concentration, Performance, prediction, Ensemble Model, XGBoost, CatBoost, LightGBM, LSTM, Bi-LSTM, GRU, ANN

## Abstract

Machine learning techniques have garnered considerable attention in modern technologies due to their promising outcomes across various domains. This paper presents the comprehensive methodology of an optimized and efficient forecasting approach for Particulate Matter 10, specifically tailored to predefined locations. The execution of a comparative analysis involving eight models enables the identification of the most suitable model that aligns with the primary research objective. Notably, the test results underscore the superior performance of an ensemble model, which integrates state-of-the-art methodologies, surpassing the performance of the other seven state-of-the-art models. Adopting a case-specific methodology with machine learning techniques contributes to achieving a notably high regression coefficient (R²≈1) across all models. Furthermore, the study underscores the potential for future endeavors in predicting location-specific environmental factors.

• This study focused on forecasting PM10 with machine learning models with the consideration of air quality factors and meteorological factors
• Ensemble model was developed for the forecasting purposes with higher performance.

## Graphical abstract

**Figure:**
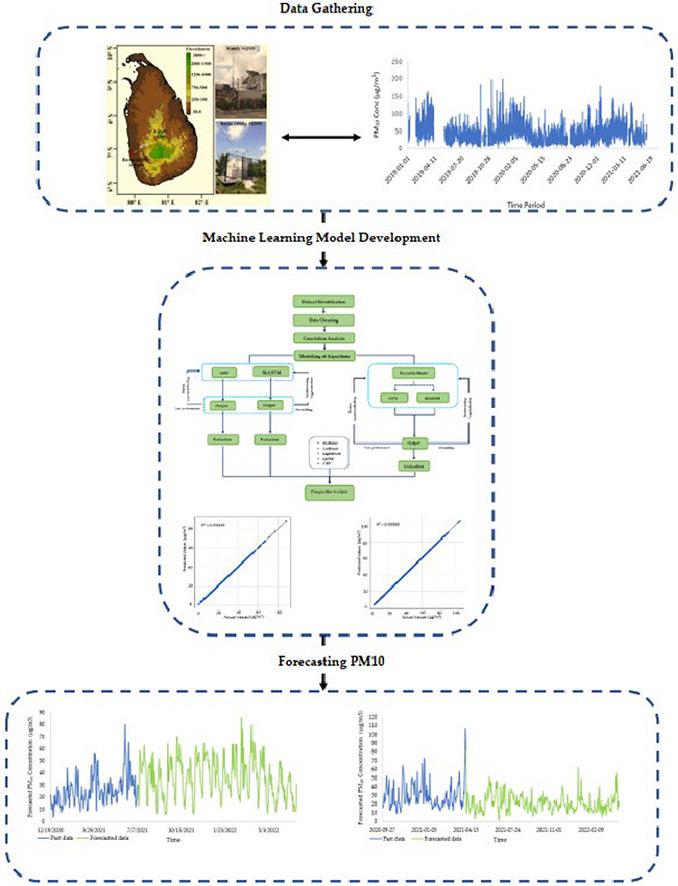

Specifications table

**Table:** 

Subject area:	Engineering
More specific subject area:	Machine Learning
Name of your method:	Ensemble Model, XGBoost, CatBoost, LightGBM, LSTM, Bi-LSTM, GRU, ANN
Name and reference of original method:	Comparative analysis of machine learning models for forecasting PM10 concentration in urban areas. Mampitiya, L.; Rathnayake, N.; Hoshino, Y.; Rathnayake, U Under review – Journal of Environmental Management
Resource availability:	The data can be requested by corresponding author only for research purposes.

**Method details**

## Background analysis

Intelligent systems are a leading component of the technological world that contributes to the development of the world. Machine Learning and artificial intelligence are some key attributes of them, where the massive workload is lying on them at present. Forecasting, Regression, and classification are some major roles played by machine learning algorithms. Patterns in between the data and correlation among data are two major elements in machine learning. The physical interpretation of machine learning techniques among data is highly important. Therefore, identifying the proper mathematical understanding of machine learning is essential. Proper structuring of the machine learning models should be carried carefully out [1].

Literature reveals that machine learning models are functioning better than statistical models in pattern identification. Therefore, machine learning models are in the spotlight due to their high performance in many fields [2], [3], [4]. Therefore, to achieve more functionality and to understand the unseen patterns, the hybrid models are developed to attract more accurate answers. Identification of the inputs and outputs of the machine learning models, with a proper methodology, should be developed at the human interaction phase of the development. Therefore, the human interaction phase of the machine learning model development is highly important.

This study presents the comprehensive methodology of several machine learning techniques in forecasting particulate matter (PM_10_) with the proper data preprocessing techniques for predefined (Kandy, Battaramulla) geographical specified areas with the analysis of meteorological data. A comparative analytical analysis was carried out under seven well-known evaluation matrices to identify the functionality of the models. The novelty and the contribution to the domain lies as this is the first study in Sri Lanka to forecast the PM10 levels for two predefined locations with a comparative analysis.

## Methodology

The steps followed in the comparative analysis using eight machine learning models are given in this section.

### Dataset preprocessing

As the initial step, the data preprocessing was carried out, to overcome the abnormalities and the misleads of the dataset. Real-world applications have many issues in missing data. To overcome the negative impact of missing and extreme data, selected impactable data were removed with the analytical help of field-related expertise [5].

Correlation of the parameters in the dataset is one of the important things that should be considered in machine learning applications. The parameter correlation was carried out to identify the main contributor. Min-Max Normalization is a technique that is applied to scale down the whole dataset to a predefined scale (0,1). Normalization of the dataset contributes to increasing the sensitivity of the data. In this study, Min-Max Normalization was carried out before feeding the data to the machine-learning models (refer to Eq. (1)).

(1) $$\text{Scaled}\;\text{Value}=\frac{\left(\text{Original}\;\text{Value}-\text{Min}\;\text{Value}\right)}{\left(\text{Max}\;\text{Value}-\text{Min}\;\text{Value}\right)}$$

### Machine learning model development

The following mathematical formulation was developed in each machine learning model (refer to Eq. (2)).

(2) $$PM_{10}=\text{Function}\left(O_{3},\;\text{CO},NO_{2},SO_{2},PM_{2.5},\;\text{AT},\;\text{RH},\;\text{SR},\;\text{RF},\;\text{WS},\;\text{WD}\right)$$

where O_3_, CO, NO_2_, and SO_2_ are ozone, carbon monoxide, nitrogen dioxide and sulfur dioxide concentrations, PM_10, and_ PM_2.5_ are particulate matter 10 and 2.5 nm, AT, RH, SR, RF, WS, and WD are ambient temperature, relative humidity, solar radiation, rainfall, wind speed, wind direction, respectively. XGBoost, CatBoost, LightGBM, LSTM, Bi-LSTM, GRU, ANN, and Ensemble models are the single and hybrid models used in this study. Since the air quality datasets are composed of seasonal changes and other undefined impacts of other different parameters, comparative analysis always leads to justified results analysis.

**Fig. 1:**
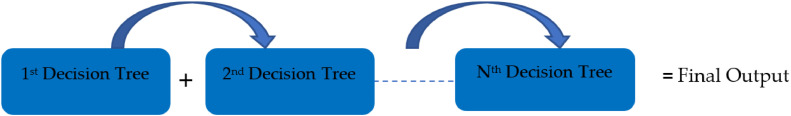
Functionality of the Decision Tree in the XGBoost algorithm.
  Eq. (3)[6] represents the objective function used in the XGBoost algorithm.
  (3) $$Obj\;\left(\theta \right)=\;\sum_{i=1}^{n}l\left(\;y_{i},\;{\hat{y}}_{i}\right)+\;\sum_{k=1}^{k}\Omega \left(f_{k}\right)\;\;$$
  Here (θ) represents the model's parameters, n is the number of samples used for training, $l(\;y_{i},\;{\hat{y}}_{i})$ is the loss function embedded in the XGBoost, while kis used as the number of trees in the ensemble model. $\Omega (f_{k})$, is the function of regularization of $k^{th}$ tree of the ensemble model [6].
(2) CatBoost – CatBoost is a powerful regression model that has a similar functionality to the XGBoost model (follows the same structure as in Fig. 1). Following the same functionality of error minimizing in XGBoost, CatBoost is a well-performing gradient-boosting algorithm in state-of-the-art models. In the domain of regression, the training of the CatBoost algorithm is fast and efficient on large datasets. Parallelization of the CatBoost model, which gives the capability of training under a few parallel ways gives the low time consumption on the training. Moreover, the development of symmetric trees’ leads to the level-by-level arrangement of the model. In addition, the feature importance selection inside the algorithm functionality contributes to selecting the best. The CatBoost algorithm also follows the objective function of XGBoost [4] (refer to Eq. (3)).
(3) LightGBM – Memory utilization is one of the underlying functionalities of the LightGBM which leads to high functionality over the different datasets. Following the histogram-based approach allows the LightGBM to achieve a higher functionality than the XGBoost. Built upon the background of the Gradient Boosting Decision Trees, LightGBM follows leaf-wise growth which helps in making the trees inside the network that leads to making the model more efficient and compact [7]. Moreover, this step of the model leads to minimizing the overfitting of the models. Additionally, LightGBM models follow a gradient-based one-side sampling technique while training to reduce the number of data points that have to be considered while training while attaining the maximum details of the data samples [5]. LightGBM gives out model output as in Eq. (4) by following the objective function.
  (4) $$Final\;Prediction=\sum_{k=1}^{k}\eta *Leaf\;Value{\;}_{k}$$
  where k is the number of trees, η is the learning rate of the model, while the $Leaf\;Value_{k}$ is the values assigned from the $k^{th}$ tree.
(4) LSTM – Long Short-Term Memory is a powerful deep learning model that follows the same structure as the RNNs. However, it has the capability of handling complex combinations of data patterns. Exploding gradients, vanishing gradients, and long-term dependencies are some examples of cases that can be undertaken by the LSTM. LSTM networks are designed with the structuring of three types of gates, including Forget Gate, Input Gate, and Output Gate. The functionality of an LSTM model can be identified in Fig. 2 [3].

(1) XGBoost – XGBoost is an algorithm that is capable of functioning well over different application datasets. The model is composed of decision trees, and they help to minimize the error at every step (refer to Fig. 1). Therefore, XGBoost has the capability of limiting its own error. Due to the optimization, and regularization techniques with the parallel performing ability, XGBoost is a well-known state-of-the-art model in regression [6].

**Fig. 2:**
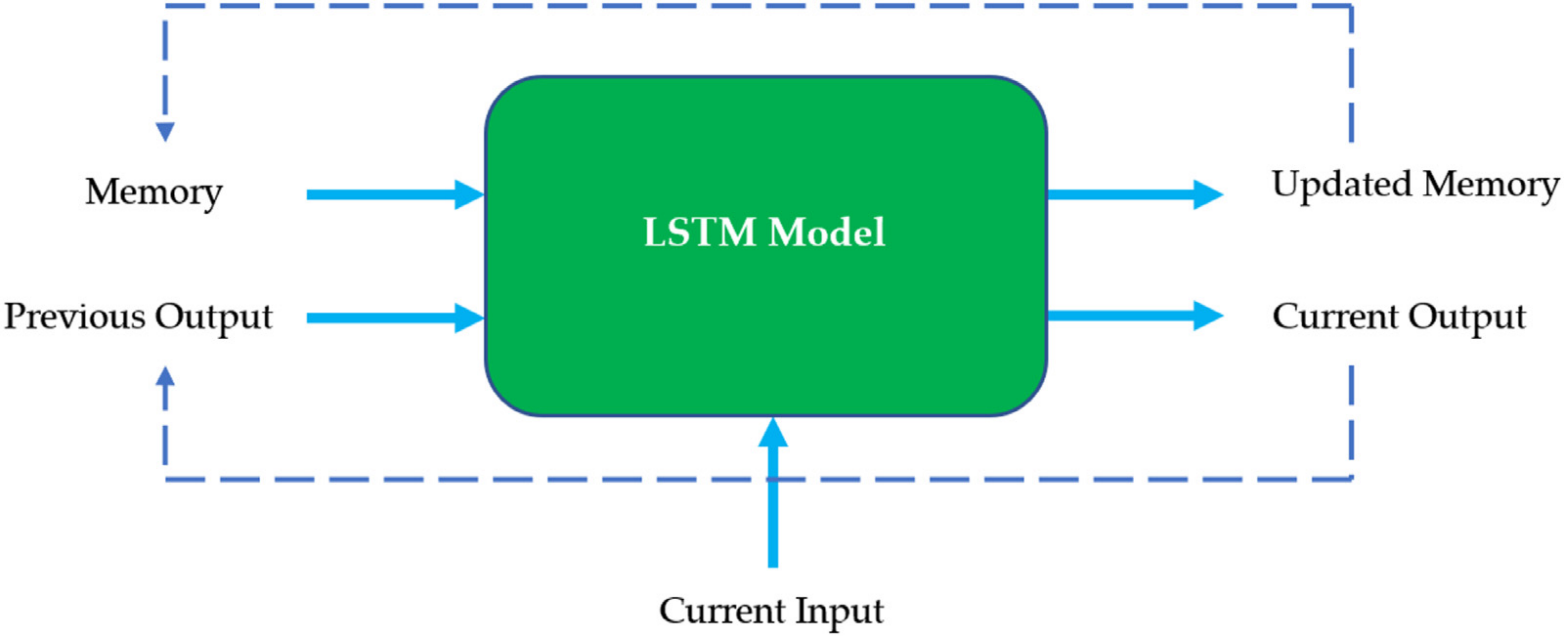
Basic functionality diagram of an LSTM.
(5) Bi-LSTM – A Recurrent Neural Network (RNN) that is capable of withstanding the noises of datasets other than the unidirectional LSTM networks. Anomaly detection and time series forecasting are the fields that Bi-LSTM plays at high-performance levels. Bi-LSTM is made up of two unidirectional LSTM networks [8]. One of them performs forward direction and the other performs in the backward direction. Since Bi-LSTM also uses the gate structures the same as in the unidirectional LSTMs and uses backpropagation through time (BPTT) it has the ability to be more powerful than the unidirectional LSTMs. One of the major concerns that was raised during this study was the computational complexity of the Bi-LSTM compared to unidirectional LSTM. The forward and backward LSTM functionality is illustrated in Fig. 3.

**Fig. 3:**
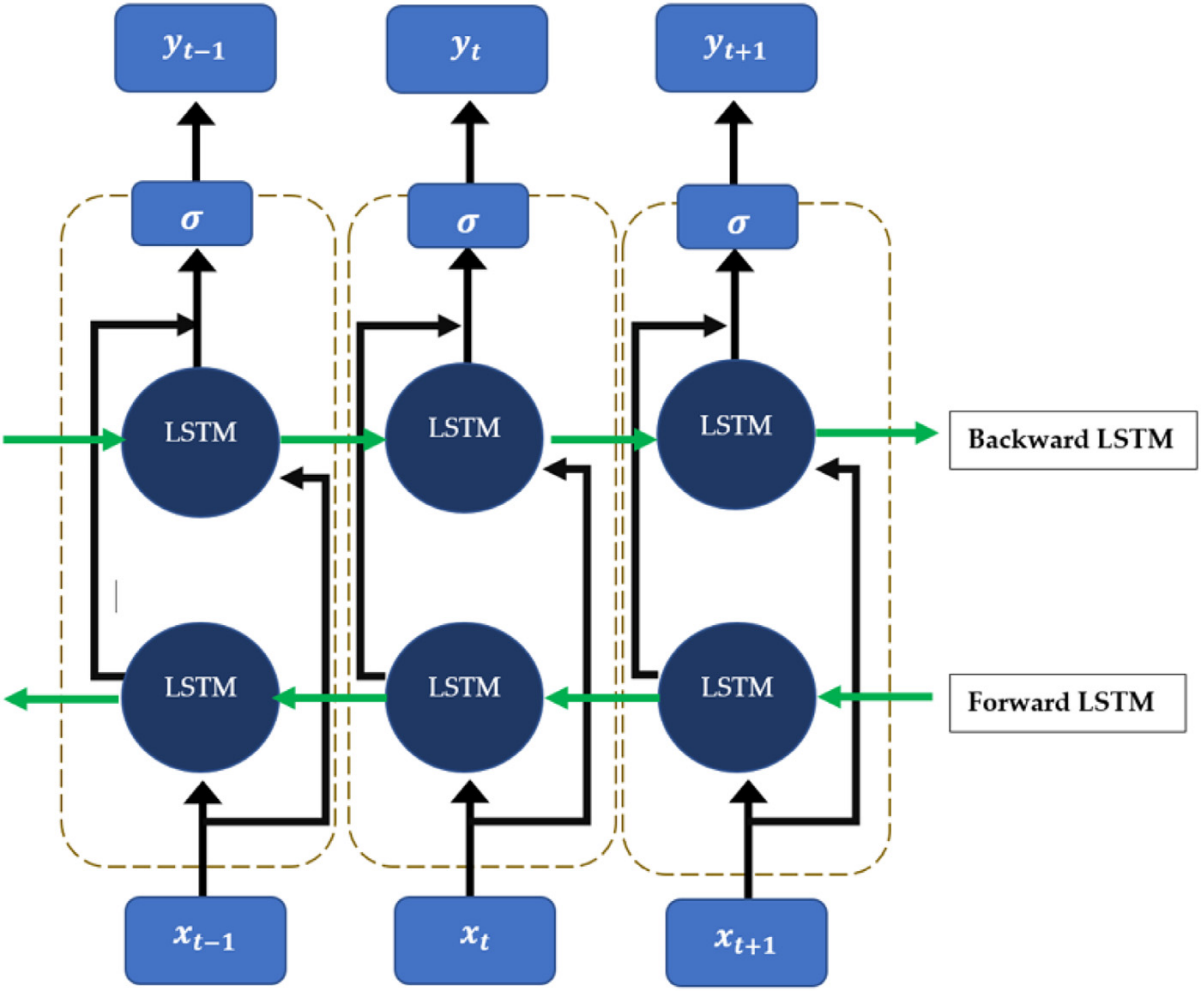
Bi–LSTM architecture with three processing levels.
(6) GRU – Gated Recurrent unit is a well-known algorithm that is used in time series analysis due to the gate structure of the model. The update grate and the reset gate are the main functioning gates of the GRU algorithm [9]. The amount of passing of the hidden state from the previous one to the next one and how much current input is used to update the hidden state are functioned by those two gates respectively followed by the sigmoid function [10]. The sigmoid function is illustratable in Eq. (5).
  (5) $$y=\frac{1}{\left(1+\;e^{-x}\right)}$$
  The functionality of the GRU model with the updating of the gates is illustrated in Fig. 4.

**Fig. 4:**
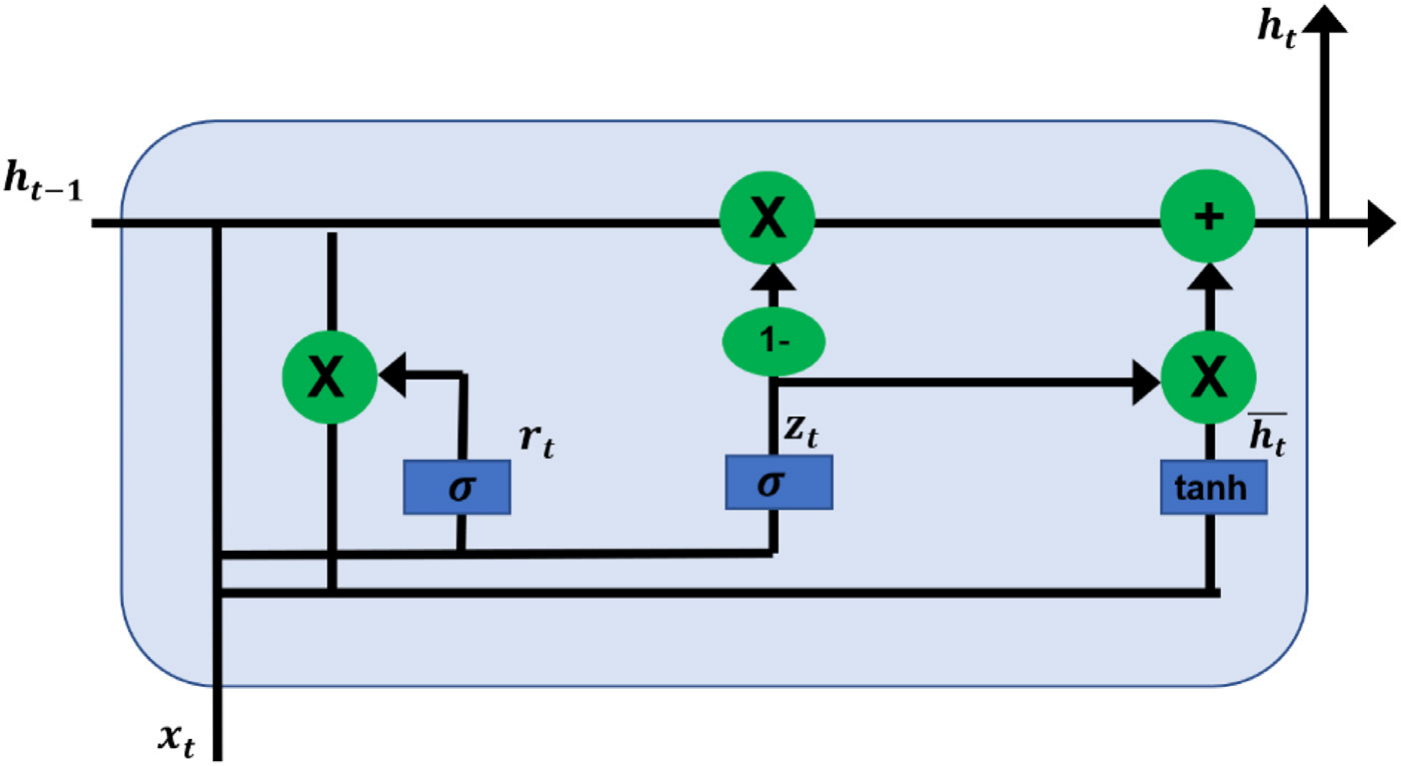
The architecture of the GRU model.
(7) ANN - Artificial Neural Networks is a state-of-the-art model that has the performability on time series analysis by understanding the complex relationships in-between the parameters. These types of networks can identify the nonlinear relationships between the input parameters and the output [11]. Moreover, when it comes to complex applications like time series forecasting, ANN is an essential factor for many researchers. Adjustability is another advantage of ANN. Fine-tuning of ANNs attain high performance. techniques like dropout and regularization make them robust to the noises of the data [12]. This makes ANNs fit well for real-world applications. The architecture that is composed of an output layer, two hidden layers, and an input layer is demonstrated in Fig. 5.

**Fig. 5:**
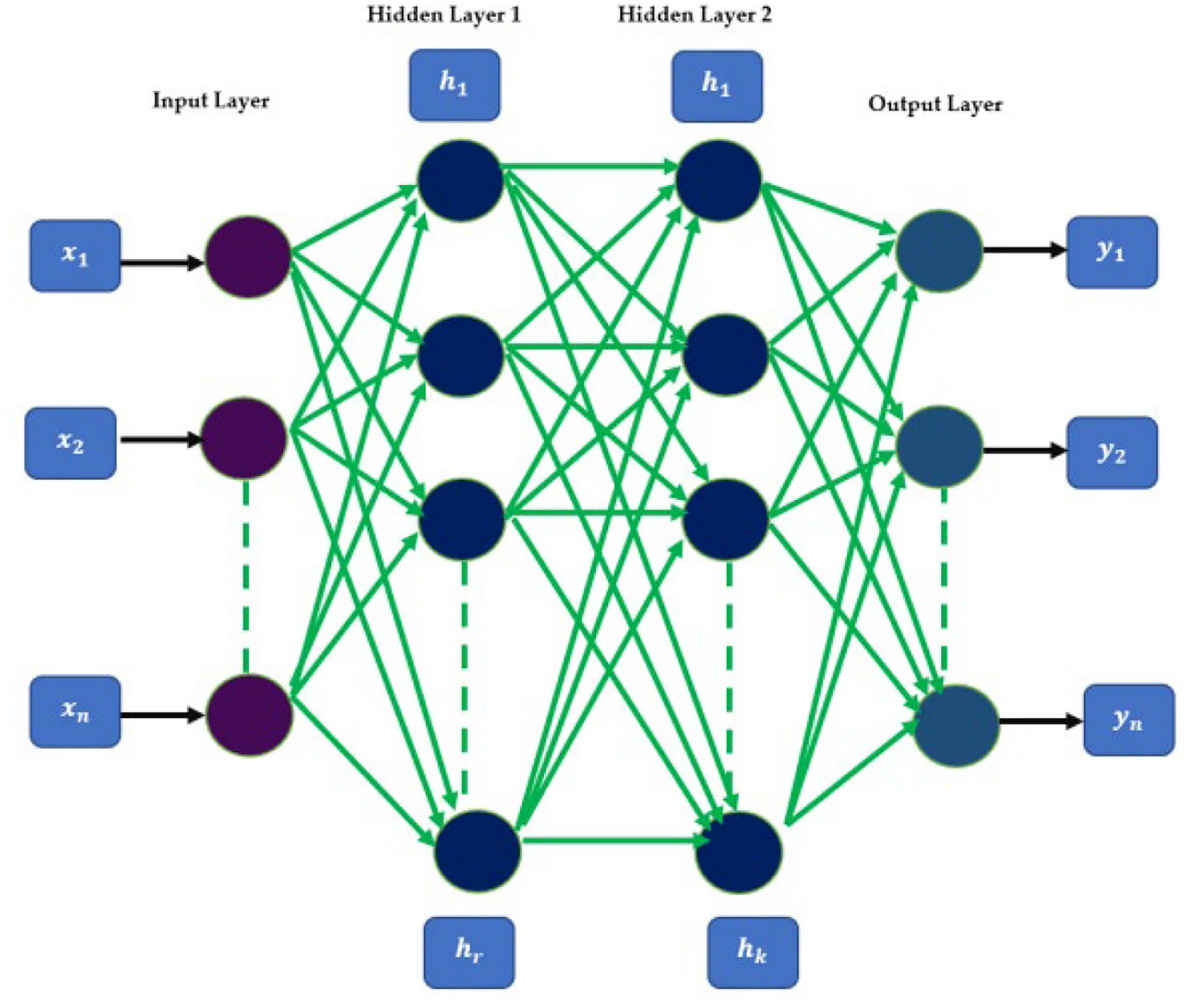
Base level architecture of ANN.
(8) Ensemble Model – In this study a manual Ensemble model was developed for the achievement of higher performance. Ensemble models are developed by a combination of selected models to work toward the same output [13]. According to the representation of Fig. 6, the ensemble model used in this study was used. Moreover, the outputs of the single models (ANN and Bi-Directional LSTM) were averaged with in order to achieve higher performance than the single models. Additionally, in the ensemble modelling, the usage of the single models were done by with the using of equal weights on both the models, (neither ANN nor Bi-Directional LSTM was manually biased). Equal weight applying toward both single models lead to treat them equally. Therefore, the averaging also carried out without considering any of the model biased for the final output.

**Fig. 6:**
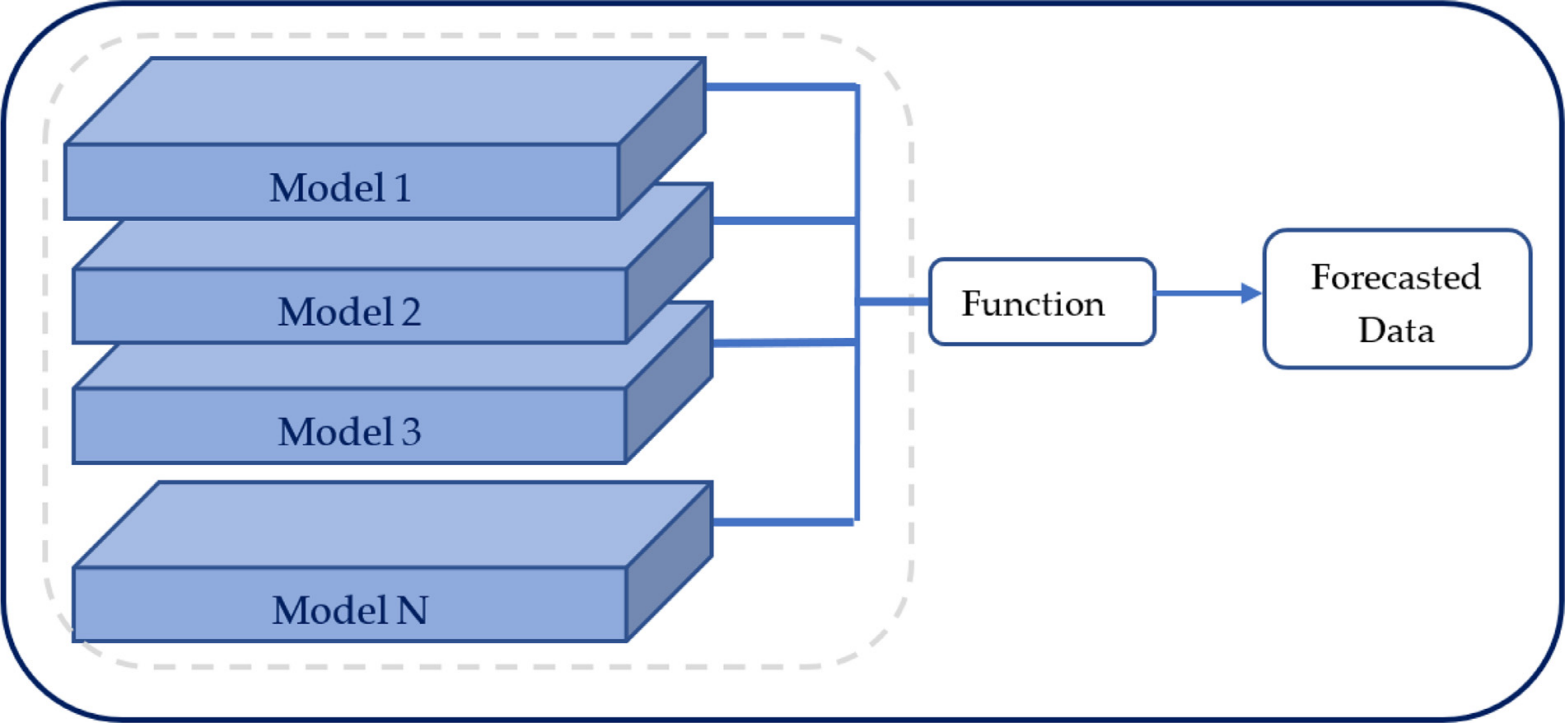
Structure of an Ensemble Model.

Ensemble models have showcased the capability of performing better than single models. The background reason behind that is, it has the capability of considering all the outputs generated from all the single models inside the ensemble model. The ensemble model was able to achieve a higher performance in forecasting by outperforming all the single models discussed, by following Eq. (6).

(6) FinalForecasting=Averagingfunction[Forecast(ANN,Bi−STM)]

The above-stated eight machine learning models were developed to mathematically formulate the nonlinear relationship showcased in Eq. (2). Proper optimization and hyperparameter tuning of the models lead to the achievement of the high performance of the models [14,15].

**Fig. 7:**
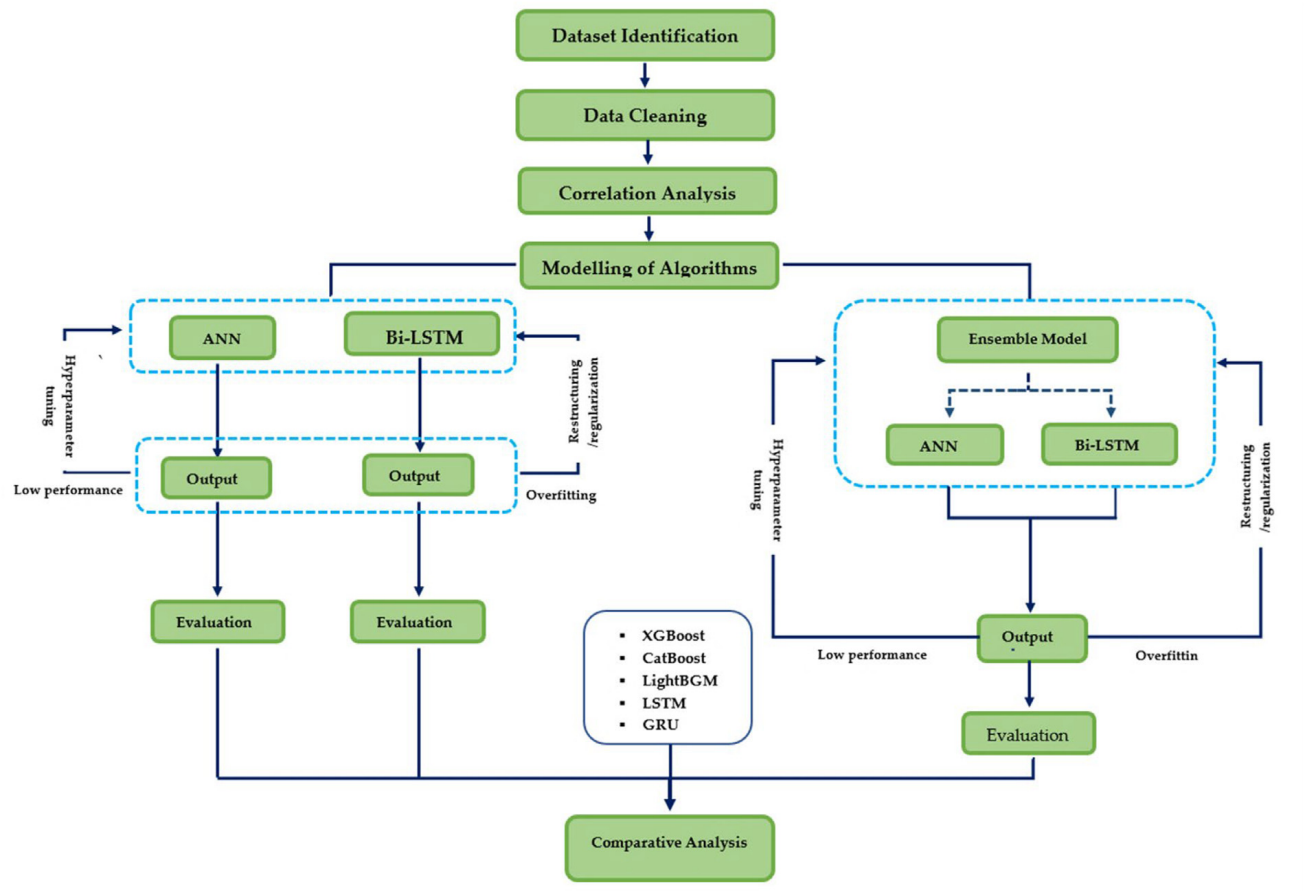
Overall methodology of the study.

After carrying out the Data Cleaning, Correlation Analysis in-between the input variables, the input data and output data were feed to developed machine learning models. With the performance evaluation of the machine learning models with respective to evaluation matrices (R^2^, RMSE, MARE, MSE, NSE, NNSE, MAE) the best performing machine learning model was chosen for the forecasting purposes. Fig. 7 presents the overall methodology carried out in forecasting the air quality.

The comparative analysis of the performance of eight machine learning models was carried out using evaluation indices such as Regression coefficient ($R^{2}$), Root Mean Squared Error (RMSE), Mean Squared Error (MSE), Mean Absolute Error (MAE), Mean Absolute Relative Error (MARE), and Nash-Sutcliffe Efficiency (NSE) were applied over the models.

The results of the study revealed that the Ensemble model is the best-suited model to predict and forecast the air quality levels in Sri Lanka. In addition, the following relationship can be presented based on the order of performance among the eight machine learning models.

(LSTM)<(XGBoost)<(GRU)<(Bi−LSTM)<(ANN)<(CatBoost)<(LightGBM)<(Ensemble)

The main objective of this study was to develop a precise model for the forecasting of air quality (PM_10_) for two locations in Sri Lanka. It was identified and concluded that the Ensemble model developed using Eq. (6), is the best-suited model. Moreover, it was concluded that the well-treating of the data and following an optimized approach will lead to the best performance of the model and make it robust. Unbiased evaluation of the models displays the correct way of analysis of the models in order to use them in real-world applications [16,17].

## Method validation

More importantly, this paper presents the comprehensive methodology carried out in the performance evaluation. The detailed results of the research work carried out can be found in Mampitiya et al. [18]. These results were obtained as an output of a research activity carried out, to forecast one of the are quality factor, PM_10_ for the predefined locations. As the output of the method validation Ensemble model developed under this research study has the capability of forecasting the PM_10_ values by outperforming other machine learning models for both locations. Furthermore, this study has only capability of functioning and forecasting the PM_10_ values only for the two predefined locations (Battaramulla and Kandy).

## Ethics statements

This work does not use the human subjects, Animals and data collected through social media as research materials.

## CRediT authorship contribution statement

**Lakindu Mampitiya:** Methodology, Software, Formal analysis, Investigation, Resources, Data curation, Writing – original draft. **Namal Rathnayake:** Validation, Resources, Writing – review & editing. **Yukinobu Hoshino:** Validation, Writing – review & editing. **Upaka Rathnayake:** Conceptualization, Resources, Writing – review & editing.

## Declaration of competing interest

The authors declare that they have no known competing financial interests or personal relationships that could have appeared to influence the work reported in this paper.

## Data Availability

Data will be made available on request.
